# Generic learning mechanisms can drive social inferences: The role of type frequency

**DOI:** 10.3758/s13421-022-01286-2

**Published:** 2022-04-14

**Authors:** Ansgar D. Endress, Sultan Ahmed

**Affiliations:** Department of Psychology, City, University of London, Northampton Square, London, EC1V 0HB UK

**Keywords:** Social learning, Language acquisition, Type frequency, Conformity, Moral decision-making, Domain generality

## Abstract

How do we form opinions about typical and morally acceptable behavior in other social groups despite variability in behavior? Similar learning problems arise during language acquisition, where learners need to infer grammatical rules (e.g., the *walk/walk-ed* past-tense) despite frequent exceptions (e.g., the *go/went* alternation). Such rules need to occur with many *different* words to be learned (i.e., they need a high *type frequency*). In contrast, frequent individual words do not lead to learning. Here, we ask whether similar principles govern social learning. Participants read a travel journal where a traveler observed behaviors in different imaginary cities. The behaviors were performed once by *many distinct* actors (high type frequency) or frequently by a *single* actor (low type frequency), and could be good, neutral or bad. We then asked participants how morally acceptable the behavior was (in general or for the visited city), and how widespread it was in that city. We show that an ideal observer model estimating the prevalence of behaviors is only sensitive to the behaviors’ type frequency, but not to how often they are performed. Empirically, participants rated high type frequency behaviors as more morally acceptable more prevalent than low type frequency behaviors. They also rated good behaviors as more acceptable and prevalent than neutral or bad behaviors. These results suggest that generic learning mechanisms and epistemic biases constrain social learning, and that type frequency can drive inferences about groups. To combat stereotypes, high type frequency behaviors might thus be more effective than frequently appearing individual role models.

**Supplementary Information:**

The online version contains supplementary material available at 10.3758/s13421-022-01286-2.

## Introduction

How do we learn about social groups, in particular about behaviors that are morally acceptable or even typical for a group? Morally acceptable behavior clearly differs across social groups and thus needs to be learned (eg., Hauser, Lee, & Huebner, 2010; Nucci & Turiel, 1978; Smetana, 1984), and attitudes and beliefs about other social groups take years to develop (Dunham, Baron, & Banaji, 2008). Further, even young children prefer the opinions or actions associated with the majority of a group (eg., Haun, Rekers, & Tomasello, 2012; Kim & Spelke, 2020), suggesting that some social learning mechanisms must be in place. Possibly, we have specifically social learning mechanisms serving as adaptations for conformity pressure (Haun et al., 2012) or norm-based group-living (Apicella & Silk, 2019).

While a notoriously social species such as humans has plausibly evolved some mechanisms to deal with social information (eg., Apicella & Silk, 2019; Dunbar, 2009; Haun et al., 2012), humans also need to learn in many other domains. It is thus possible that humans learn effectively about other social groups, but using generic learning mechanisms that are not specifically social and similar to those found in other domains such as language acquisition.^1^ We focus on learning which behaviors are morally acceptable in, or even typical of, a social group. Specifically, we ask which cues observers use to estimate (i) the prevalence and (ii) the moral acceptability of behaviors. While prevalence and moral acceptability might sometimes be related (e.g., severe moral transgressions like murder might be rare *because* they are transgressions), they can also be independent (e.g., costly prosocial behaviors such as giving to charity might be praiseworthy irrespective of their frequency); we thus independently assess estimates of prevalence and acceptability.

Learners must determine acceptable or typical behavior despite the presence of inevitable “exceptions” who behave in atypical ways. Similar learning problems occur in numerous cognitive domains, from categorization (eg., Erickson & Kruschke, 1998; Nosofsky, Palmeri, & McKinley, 1994) to reading (eg., Coltheart, Rastle, Perry, Langdon, & Ziegler, 2001; Miceli, Capasso, & Caramazza, 1994) to grammar (eg., Pinker, 1999). For example, grammatical regularities like the English past-tense have regular alternations (e.g., walk/walk-ed) and exceptions to this alternation (e.g., go/went). Different authors suggested that, to learn regular alternations, the alternations need to have high *type frequencies*, that is, they needs to occur with many *different* words. In contrast, to learn exceptions, they need to have high *token frequencies*, that is, they need to occur sufficiently often (e.g., Baayen & Lieber 1991; Bybee 1995; Dabrowska & Szczerbiński 2006; Endress & Hauser 2011; Marchman & Bates 1994, but see Marcus, Brinkmann, Clahsen, Wiese, & Pinker, 1995). This is clearly the case for the English past-tense: exceptions like go/went are among the most frequent English words (i.e., they have high token frequencies), while the regular past-tense applies to a huge number of words (i.e., they have high type frequencies), even though most words are exceedingly rare (Yang, 2013).

Here, we ask whether similar learning principles apply in the social domain. Are observers sensitive to the type frequency of behaviors when judging their typicality or acceptability? In other words, do they take into consideration the number of *distinct* individuals performing a behavior, or do they just consider the raw frequency with which a behavior is observed? For example, to reduce persistent science-related gender stereotypes (Nosek et al., 2009), are media reports more effective if they report on few but highly visible female scientists (e.g., Nobel laureates, who appear (relatively) frequently in the media and thus have high token frequencies, but low type frequencies), or rather if they report on more numerous but less prominent scientists (who appear rarely in the media and thus have low token frequencies, but (relatively) higher type frequencies)?

We are certainly not the first ones to suggest that research on language acquisition might inform research on moral development. Different authors suggested that humans might be equipped with a moral faculty akin to the language faculty (eg., Dwyer, Huebner, & Hauser, 2010; Hauser, Young, & Cushman, 2008; Hauser, 2006; Mikhail, 2007), that is, a corpus of innate and largely unconscious knowledge that, over development, becomes tuned to the specific linguistic or cultural environment a learner happens to grow up in. If so, mechanisms used in language acquisition might also contribute to moral and social development. Likewise, if moral development amounts to learning categories such as “good” and “bad” (McHugh, McGann, Igou, & Kinsella, 2021), the fact that learners need to induce categories in the presence of exceptions (Erickson & Kruschke, 1998; Nosofsky et al., 1994) suggests that type frequency might also affect the learning of moral categories.^2^

As mentioned above, there is some evidence for a sensitivity to type frequency in the social domain. For example, even when exposed equally often to different actions or opinions, young children prefer those actions or opinions associated with the majority of a group (Haun et al., 2012; Kim and Spelke, 2020), and thus the actions or opinions with the highest type frequency. While such preferences have been explained with specifically social factors like conformity biases (Haun et al., 2012), it is unclear to what extent conformity really drives behavioral choices, especially across cultures (Bond & Smith, 1996). Even in Asch (1951) classic line-comparison task, 26% of the participants showed no conformity bias whatsoever, and, across participants, 68% of the trials showed no sign of conformity (see Corriveau and Harris (2010) for similar results with children). In other cases, it is unclear how group-relevant conforming behavior can even be learned, especially in the case of *prohibitions*. For example, picking one’s nose and singing Don Giovanni’s Champagne Aria are unusual dinner table behaviors, but only the former is culturally prohibited, and, in the absence of explicit admonitions, learners cannot interpret the mere absence of behaviors as evidence for prohibitions (see Gleitman & Wanner, 1982, for related problems in language acquisition). Conversely, overt conformity is unlikely to have evolved as a reliable signal of group-membership. After all, wholesale copying of overt behaviors is easy to fake and thus not an evolutionarily stable signal (Zahavi, 1977).

Alternatively, majority-based choices might reflect epistemic biases (Kim & Spelke, 2020), perhaps because majority opinions are perceived as more veridical (e.g., due to wisdom of the crowd effects; Galton, 1907), or, if shared cultural knowledge indicates group-membership (Soley & Spelke, 2016), because majority behaviors are better cues to shared cultural traditions. Accordingly, majority-based choices can be overridden when better information sources are available (Burdett et al., 2016; Kim & Spelke, 2020; Wilks, Collier-Baker, & Nielsen, 2015).

If majority-based choices reflect epistemic biases, learners might well apply the same learning principles to social information that they apply to other kinds of information, and might be sensitive to the type frequency of actions in the complete absence of conformity or other social pressures.^3^

To test this idea, participants read a cover story of a traveler (Noah) spending 10 days in each of several imaginary cities. On each day of his visit, Noah observed a behavior that was either performed by the same individual ten times or once by ten different individuals; the behavior was morally good, neutral, or bad. Across participants, we also manipulated the gender of the person performing the behavior. For example, in (the imaginary city of) Elder, Noah might see the same women pantomiming in the station on all 10 days of his visit, or see a different women pantomiming on each of the 10 days. As a result, the number of occurrences of the behavior was equated but the type frequency was higher in the latter case. Critically, the stories pragmatically implied that there were other individuals *not* engaging in the behavior (otherwise, a universal quantifier like *all* would have been used; see Chierchia (2004), for a discussion of scalar implicatures); as a result, participants had to detect typical behavior despite the presence of individuals not engaging in the behavior.

After each scenario, we asked participants five questions, related to (1) how morally acceptable the behavior was for the participants themselves (first-party acceptability), (2) how morally acceptable it was to other habitants of the imaginary city of the same gender as the actor (e.g., other women in Elder; gendered third-party acceptability), (3) how morally acceptable it was to people in the imaginary city *in general* (e.g., people in Elder in general; general third-party acceptability), (4) how likely other habitants of the same gender were to perform the behavior (gendered behavior prevalence), and (5) how likely other habitants *in general* were to perform the behavior (general behavior prevalence).

To assess the role of *type frequency*, we compared ratings in situations where the behavior was performed once by ten different individuals (high type frequency) to ratings in situations where the behavior was performed ten times by the same individual (low type frequency); to assess the role of the *valency* of the behavior, we compared ratings for behaviors that differed in valency. We expected that the type frequency of a behavior would be positively related to judgements of third-party acceptability and prevalence, and potentially also to first-party acceptability. While we expected first- and third-party acceptability judgements to mirror the valency of behaviors, we asked whether the effects of *type frequency* and *valency* would interact. If the effects interact, the effect of *type frequency* might be most pronounced for neutral behaviors rather than intrinsically good or neutral behaviors; alternatively, the effects of *type frequency* and *valency* might also be additive.

Before presenting the results, we show that a selective sensitivity to type frequency is also expected from a fairly generic ideal observer model trying to maximize reward likelihoods based on discrete observations.

## An ideal-observer model to determine typical behavior

Humans need to *generalize* representative patterns in a population for a variety of reasons. In the non-social examples above, they need to separate grammatical rules from exceptions, orthographic regularities from idiosyncratic spellings, or diagnostic object features from irrelevant features. In the social domain, learners need to track representative behaviors because majority behavior might reflect accurate knowledge about resources, because majority behavior is less likely to lead to punishment (e.g., in the case of moral transgressions), because coordination problems like cooperative hunting or anti-predator defense require behavior that is consistent in a population, or for any number of other reasons. If humans have a need for belonging (Baumeister and Leary, 1995), conformity biases might be a special case of reward seeking (or punishment avoidance) by maximizing the chance of feeling part of a group.

We thus develop a simple Bayesian decision model, where observers witness a behavior *B*, potentially performed *k* times ${b_{i}^{a}}$ (*i* = 1…*k*) by actor *a*. They then need to decide if actors are typical examples of the group in terms of their susceptibility to engage in a behavior, or if they are exceptions. Observers assume that actors behave *consistently*. That is, if actors have been observed engaging in a behavior, observers assume that the actors are susceptible to engage in it at a later time. This assumption simply reflects the finding that humans tend to explain actions by attributing consistent dispositions to actors, even when these explanations are not particularly veridical (eg., Jones & Nisbett, 1972; Nisbett, Caputo, Legant, & Marecek, 1973; Watson, 1982). Such attributions are unlikely to be specifically social; after all, observers likely assume that the same banana will have the same sweet taste upon repeated tasting, even though the taste is just as invisible as an attributed disposition. In line with this view, adults and children attribute consistent dispositions in the form of essentialist beliefs not only to biological and non-biological objects in general (eg., Gelman, 2003; Medin & Ortony 1989), but also hold essentialist beliefs about social and behavioral traits in people (eg., Dar Nimrod & Heine, 2011). Adults and children also hold essentialist beliefs about the moral character of actors (eg., Heiphetz, 2020). For example, moral judgments are affected by judgments about the moral character of an actor (Siegel, Crockett, & Dolan, 2017; Uhlmann, Pizarro, & Diermeier, 2015), and both children and adults attribute “goodness” to an internal, unchanging “essence” of an actor (Heiphetz, 2019).

If observers attribute consistent dispositions to actors, the probability for an actor to be susceptible to engaging in a behavior is independent of the number of times it is performed by the actor. This can be formally seen by using the chain rule on $P({b_{1}^{a}},\ldots , {b_{k}^{a}} | \text {actor})$, that is, the probability that actor *a* is susceptible to engage in a behavior *B* *k* times:

$$\begin{array}{@{}rcl@{}} P({b_{1}^{a}},\ldots, {b_{k}^{a}} | \text{actor}) & =& P({b_{k}^{a}} | {b_{1}^{a}} {\dots} b_{k-1}^{a}, \text{actor}) \\ && \times P(b_{k-1}^{a} | {b_{1}^{a}} {\dots} b_{k-2}^{a}, \text{actor}) \times {\ldots} \\ && \times P({b_{2}^{a}} | {b_{1}^{a}}, \text{actor}) \times P({b_{1}^{a}} | \text{actor}) \end{array}$$

As actors are assumed to act consistently, all terms but the last one are equal to one, and the likelihood of an actor being susceptible to perform a behavior *k* times is identical to the likelihood of an actor to be susceptible to perform it once, that is $P({b_{1}^{a}} | \text {actor})$. In other words, if observers treat people like bananas (or other objects), and explain behavior by attributing consistent dispositions to actors, they should be relatively insensitive to the token frequency of a behavior, and repeated performance of the same action by the same actor should be relatively uninformative.

That being said, estimates of behavior prevalence might still be sensitive to token frequency, for example if the token frequency needs to be sufficiently high so that dispositions can be *remembered* (see Endress & Hauser, 2011); high token frequency individuals might also affect views about groups due to the availability heuristic (Tversky & Kahneman, 1973), as they are more likely to come to mind than low token-frequency individuals. However, in the absence of such memory retrieval issues, the token frequency of a behavior should be relatively uninformative about the dispositions causing this behavior.

As some behaviors are inherently weirder than others, we further assume that observers have some prior beliefs about the typicality of the behavior, and that behavior *B* has a prior probability *P*(*B*) = *β*. Observers also have prior beliefs about the proportion of exceptions *𝜖* in the group. This leads to two hypotheses, according to which the actor is (1) typical of a group or (2) an exception.

First, according to *H*_1_, the actor is typical of a group that engages in the behavior with a large probability *α* to begin with. If so, the probability of observing the behavior is identical irrespective of whether the actor is typical or an exception *P*(*B*|typical) = *P*(*B*|exception) = *α*. Second, according to *H*_2_, the actor is an exception. If so, the probability of observing the behavior if the actor is an exception is *P*(*B*|exception) = *α*, while the probability of observing the behavior in the general population is just its prior (and lower) frequency *P*(*B*|typical) = *β* < *α*.

Following Tenenbaum and Griffiths (2001), one can assume that both hypotheses are equally likely a priori, and evaluate the evidence in favor of *H*_1_ by comparing the likelihood ratio

$${\Lambda}_{1,2} = \frac{P(B|H_{1})}{P(B|H_{2})}$$

Assuming that the behavior is observed with *T* different actors, this likelihood ratio becomes

$$\begin{array}{@{}rcl@{}} {\Lambda}_{1,2} & = & \frac{\left( (1-\epsilon) \alpha + \epsilon \alpha\right)^{T}}{\left( (1-\epsilon) \beta + \epsilon \alpha\right)^{T}} \\ & = & \frac{1}{\left( (1-\epsilon) \frac{\beta}{\alpha} + \epsilon\right)^{T}} \\ & \approx & \left( \frac{\alpha}{\beta}\right)^{T} \left( 1 - T \left( \frac{\alpha}{\beta} -1 \right) \epsilon \right) \end{array}$$

where the last step is the Taylor expansion for small values of *𝜖*.

**Fig. 1:**
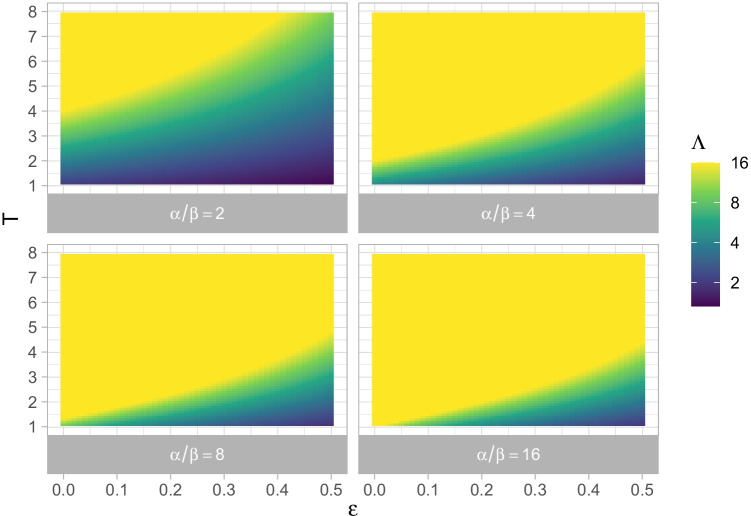
Likelihood ratio in favor of the hypothesis that a behavior is representative of a population (rather than an exception) as a function of the prior frequency of exceptions (*𝜖*), the type frequency (*T*) and the ratio *α*/*β* (facets)

An illustration for some values of the *β*/*α* ratio are given in Fig. 1. As $\frac {\beta }{\alpha } (1-\epsilon ) + \epsilon$ is strictly smaller than 1 for *α* > *β* and *𝜖* < 1, the likelihood ratio is always greater than 1, and grows exponentially as the type frequency increases. In other words, the model suggests that behaviors with higher type frequencies should be considered more representative.

In the Supplementary Online Material SOM1, we also use Bayes’ rule to directly calculate the posterior probability *P*(*H*_1_|*B*) of a behavior being typical of a population while assuming that the prior probability of the actor being an exception is given by the prior probabilities of exceptions. We show that it is approximately $1 - \epsilon \left (\frac {\beta }{\alpha }\right )^{T}$, and thus exponentially converges to one as the type frequency of the actions increases. This confirms that, for an ideal observer model explaining behavior with consistent dispositions, the estimates of typicality grow exponentially with the type frequency of the behavior.

## Methods

### Participants and sample size

Participants in the main experiment were recruited through testable minds (http://minds.testable.org) and were paid $5.5 for their time. Participants were excluded from analysis if (1) they did not complete the experiment, (2) did not pass the attention check (i.e., did not remember the name of the traveler), or (3) always gave the same response. In total, 144 participants were retained for analysis in the main experiment (69 females, 72 males, 3 non-identified, mean age 32.3 y).

The sample size was chosen to achieve 80% power for a weak interaction between *question generality*, *valency* and *gender match* observed in the pilot experiment reported in Supplementary Information SOM3 (see below for a definition of these predictors); in that study, 90 participants (81 females, 9 males, mean age 19.2 years) were retained for analysis from the City, University of London study pool and received course credit for their time. It turned out, however, that this interaction was an artifact of choosing an inappropriate base-level for *valency* in the generalized linear mixed models reported below, and disappeared once neutral actions were used as a base-level. Otherwise, the results were essentially identical to those of the main experiment.

### Materials

Participants were first presented with the following cover story.

Noah is studying journalism at university. An opportunity comes up where he decides to take a gap year to explore some countries and cities that have always interested him.

He usually stays 10 days in each place. In each place, Noah makes some interesting observations.

Following this, they were asked about the name of the traveler (as an attention check) and then proceeded to eight scenarios with good, neutral and bad actions presented in random order (24 scenarios in total, see Supplementary Online Material SOM5) occurring in imaginary cities. The city names were generated from https://fantasynamegenerators.com. An example of a neutral scenario as well as its associated questions is given below:

*Low type frequency version*: In Qrita, Noah saw the same woman at the bus stop every day of his 10-day stay and she would always wear sunglasses.

*High type frequency version*: In Qrita, Noah saw a different woman at the bus stop every day of his 10-day stay and they would all wear sunglasses.

- How likely are *women* in Qrita to wear sunglasses?
- How likely are people in Qrita *in general* to wear sunglasses?
- Would *women* in Qrita consider it morally acceptable to wear sunglasses?
- Would people in Qrita *in general* consider it morally acceptable to wear sunglasses?
- Would *you* consider it morally acceptable to wear sunglasses?

Each question was answered on a four-point-scale. The behaviors in the scenarios were selected to avoid floor and ceiling effects for moral acceptability and prevalence judgements. As a result, care was taken that the behaviors were unusual but plausible; for good and bad behaviors, they were selected to have clear valency without being extreme behaviors. The scenarios were presented through a Qualtrics survey (https://www.qualtrics.com).

### Counterbalancing

For each participant, 12 scenarios were selected to occur in the high type frequency version and 12 in the low type frequency version; this assignment was approximately counterbalanced across participants. Within each of these groups, half of the participants read scenarios with female actors, and half with male actors.

### Analysis

Given that the data were not normally distributed (see Supplementary Online Material SOM2), we converted the trial-by-trial responses to a binary random variable (coding ratings of 1 and 2 as negative, and ratings of 3 and 4 as positive). We then analyzed the trial-by-trial data using generalized linear mixed models for binary data. In addition to the factors outlined above, we also asked if participants would be more likely to generalize to individuals of the same gender rather than across genders (*question generality*), and if their ratings depended on whether their own gender matched that of the actors (*gender match*).

For each analysis below, we first fitted a model with the fixed factor predictors *question generality* (gendered vs. general; base-level: general), *type frequency* (high vs. low; base-level: high), *valency* (good vs. neutral vs. bad; base-level: neutral) and *gender match* (match vs. mismatch; base-level: match), their interactions as well as the random intercepts for participants and scenarios. Following Baayen, Davidson, and Bates (2008), we then removed those predictors not contributing the model likelihood. Both random factors contributed to the model likelihood in all analyses below. The complete results of the GLMMs are shown in Table 1; only significant predictors are mentioned in the main text.

## Results

### First-party acceptability

**Fig. 2:**
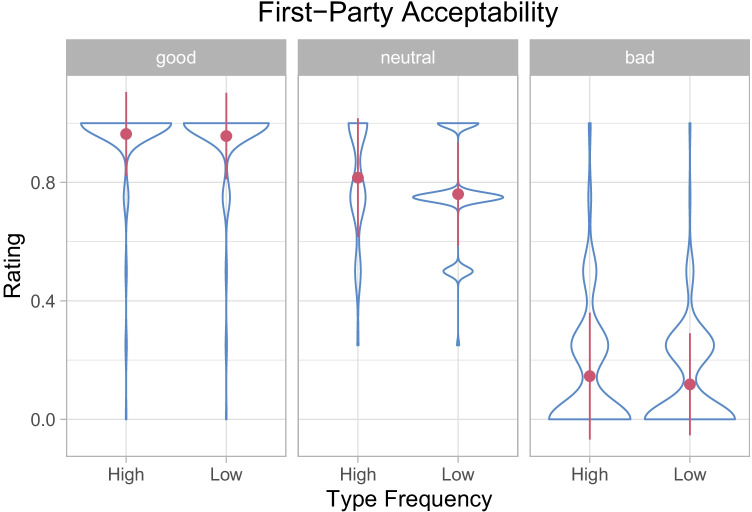
First-party acceptability as a function of valency and type frequency. The *contours* represent the distribution of responses, the *dots* the sample averages, and the *error bars* the standard deviations

The first analysis focused on first-party acceptability, that is, the degree to which the participants *themselves* considered a behavior morally acceptable. The results are shown in Tables 1 and 2 as well as Fig. 2. (Further descriptives are found in Supplementary Online Material SOM4).

Good behaviors were rated as much more acceptable than neutral behaviors, which were rated much more acceptable than bad behaviors. In other words, the participants’ first-party acceptability judgements validated our valency manipulation.

Critically, ratings were higher in the high type frequency condition than in the low type frequency condition, suggesting that the number of *different* individuals performing a behavior (rather than the raw frequency of a behavior) affects its moral acceptability even when observers just read about hypothetical scenarios.

In the pilot experiment, we observed an interaction between *type frequency* and *valency*. Follow-up GLMMs revealed that the effect of *type frequency* was more pronounced for neutral scenarios than for other scenarios. While the interaction did not reach significance in the main experiment, the effect of *type frequency* was numerically much more pronounced in the neutral condition than in the other conditions and reached significance only in the neutral condition (see Table 1). It thus seems that intrinsically neutral behaviors can acquire moral valency simply by virtue of being performed by multiple actors, while moral evaluations of behaviors with intrinsic valencies seem to be less affected by the type frequency of the behaviors.

### Third-party acceptability

**Fig. 3:**
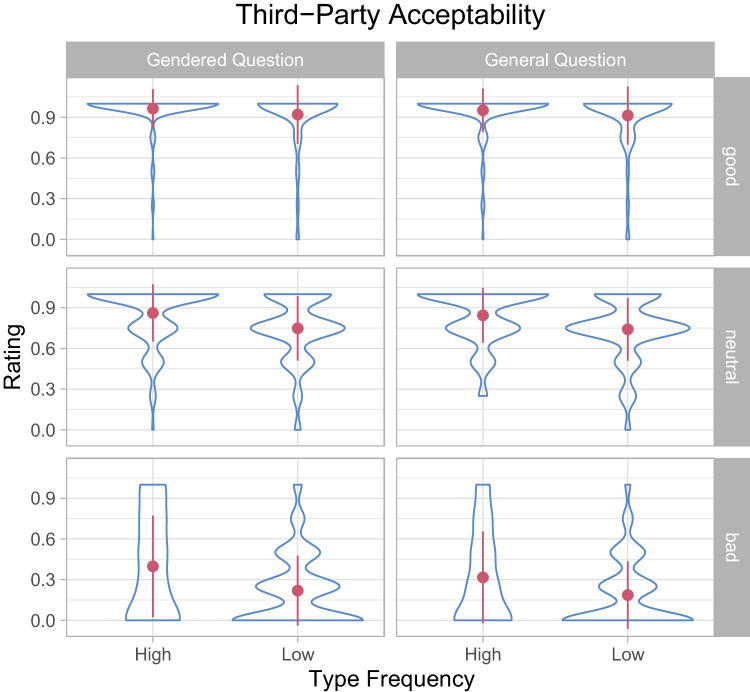
Third-party acceptability as a function of valency and type frequency. The *contours* represent the distribution of responses, the *dots* the sample averages, and the *error bars* the standard deviations

We next explored the determinants of third-party acceptability, that is, the perceived moral acceptability of behaviors for habitants of the imaginary city in a scenario. As mentioned above, we asked this question both in a gendered and a non-gendered way. That is, participants were asked (1) how acceptable a behavior would be *for other (wo)men* in the imaginary city when the behavior was performed by a (wo)man, and (2) how acceptable the behavior would be for habitants of the imaginary city *in general*. The results are shown in Tables 1 and 2 as well as in Fig. 3.

As for first-party acceptability, third-party acceptability ratings were higher for good behaviors than for neutral behaviors, and higher for neutral behaviors than for bad behaviors. Critically, third-party acceptability ratings were higher for high type frequency behaviors than for low type frequency condition behaviors, suggesting that participants believe that the number of *different* individuals performing a behavior (rather than the raw frequency of a behavior) reflects its moral acceptability in a community.

### Behavior prevalence

**Fig. 4:**
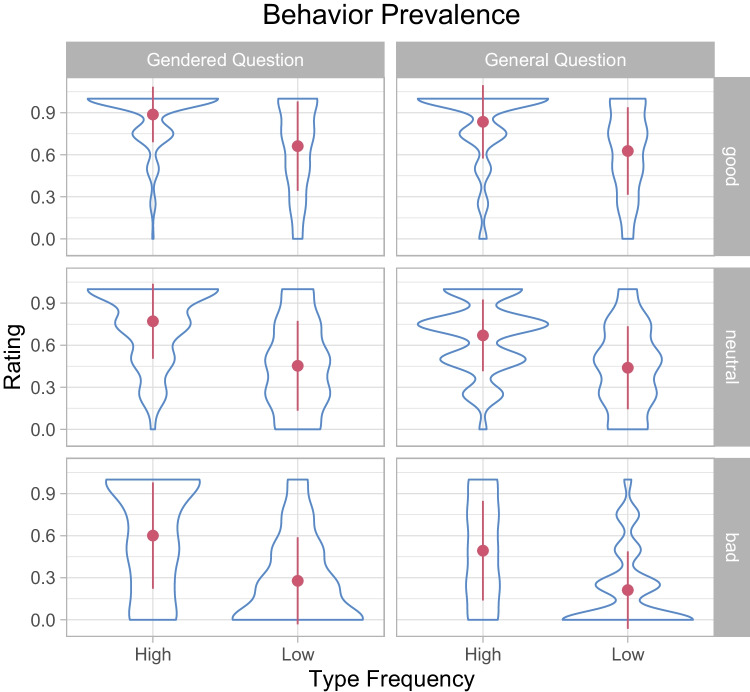
Behavior prevalence as a function of valency, type frequency, and question generality (gendered vs. general question). The *contours* represent the distribution of responses, the *dots* the sample averages, and the error bars the standard deviations

We finally explored the determinants of perceived behavior prevalence, that is, the belief that other members of a group (i.e., other habitants of the city in the scenario) would engage in a behavior. As mentioned above, we asked this question both in a gendered and a non-gendered way. That is, participants were asked (1) how likely *other (wo)men* in the imaginary city would be to perform a behavior when it was performed by a (wo)man, and (2) how likely people in the imaginary city *in general* would be to perform the behavior. The results are shown in Tables 1 and 2 as well as in Fig. 4.

The prevalence of good behaviors was rated higher than that of neutral behaviors, which was rated higher than that of bad behaviors. Critically, the prevalence was rated higher for high type frequency behaviors than for low type frequency behaviors, suggesting that participants use the number of *different* individuals performing a behavior (rather than its raw frequency) as an indicator of behavior prevalence.

Finally, the prevalence ratings were somewhat higher for the gendered question than for the general question, suggesting that participants were more likely to generalize a behavior within genders than across genders. An interaction with type frequency suggested that this effect was less pronounced in the low type frequency condition, presumably because prevalence ratings were lower in the low type frequency condition to begin with.

The match between the participant gender and that in the actor in the scenario and its interactions with other predictors did not contribute to the model likelihood.

Finally, participants might have a conformity bias, and might have might have treated the task as conformity judgement task. That is, they might have used the information in the scenarios to judge whether the behavior in the scenarios conforms to a hypothetical majority behavior. Critically, if participants had a conformity bias, they should assume that *all* behaviors are representative of the general population, including those presented in the low type frequency condition. In contrast, as shown in Table 2, they considered low type frequency behavior as *unrepresentative* of the population. As a result, participants do not seem to hold the default assumption that actions show conformity with the wider population, and require additional evidence (e.g., from type frequency) to conclude that a behavior might be representative of the wider population.

**Table 1:** Results of generalized linear mixed models of the trial-by-trial data for first-party acceptability, third-party acceptability and behavior prevalenc﻿e. See analysis section for the model specification

Effect	*β*	*SE*	*CI*	*t*	*p*
**First-party acceptability - Overall GLMM**					
Type frequency: Low	− 0.525	0.177	− 0.873, − 0.178	− 2.962	0.003
Valency: Good	1.495	0.605	0.31, 2.68	2.472	0.013
Valency: Bad	− 4.503	0.581	− 5.64, − 3.36	− 7.753	0.000
Type frequency: Low × Valency: Good	0.272	0.344	− 0.402, 0.946	0.792	0.428
Type frequency: Low × Valency: Bad	0.223	0.257	− 0.282, 0.728	0.866	0.386
**First-party acceptability - Neutral valency only**					
Type frequency: Low	− 0.545	0.179	− 0.896, − 0.194	− 3.045	0.002
**First-party acceptability - Neutral valency excluded**					
Type frequency: Low	− 0.218	0.315	− 0.834, 0.399	− 0.692	0.489
Valency: Bad	− 5.992	0.458	− 6.89, − 5.09	− 13.075	0.000
Type frequency: Low × Valency: Bad	− 0.052	0.368	− 0.774, 0.669	− 0.142	0.887
**Third-party acceptability**					
Question generality: General	− 0.224	0.153	− 0.523, 0.0751	− 1.468	0.142
Type frequency: Low	− 1.087	0.150	− 1.38, -0.793	− 7.236	0.000
Valency: Good	1.260	0.466	0.346, 2.17	2.703	0.007
Valency: Bad	− 3.228	0.439	− 4.09, − 2.37	− 7.359	0.000
Question generality: General × Type frequency: Low	0.192	0.150	− 0.102, 0.485	1.280	0.200
Question generality: General × Valency: Good	− 0.089	0.219	− 0.519, 0.341	− 0.404	0.686
Question generality: General × Valency: Bad	− 0.231	0.165	− 0.554, 0.0933	− 1.395	0.163
Type frequency: Low × Valency: Good	0.161	0.228	− 0.285, 0.608	0.708	0.479
Type frequency: Low × Valency: Bad	0.037	0.167	− 0.291, 0.365	0.221	0.825
**Behavior prevalence**					
Question generality: General	− 0.536	0.135	− 0.8, − 0.272	− 3.981	0.000
Type frequency: Low	− 1.808	0.138	− 2.08, − 1.54	− 13.141	0.000
Valency: Good	0.982	0.406	0.186, 1.78	2.419	0.016
Valency: Bad	− 1.021	0.398	− 1.8, − 0.241	− 2.567	0.010
Gender match: Mismatch	0.176	0.231	− 0.277, 0.629	0.760	0.447
Question generality: General × Type frequency: Low	0.341	0.123	0.0996, 0.582	2.769	0.006
Question generality: General × Valency: Good	0.001	0.153	− 0.299, 0.301	0.008	0.994
Question generality: General × Valency: Bad	− 0.113	0.144	− 0.396, 0.17	− 0.781	0.435
Type frequency: Low × Valency: Good	0.069	0.158	− 0.241, 0.378	0.434	0.664
Type frequency: Low × Valency: Bad	− 0.011	0.146	− 0.298, 0.276	− 0.077	0.938
Question generality: General × Gender match: Mismatch	0.001	0.122	− 0.237, 0.239	0.009	0.993
Type frequency: Low × Gender match: Mismatch	0.037	0.126	− 0.21, 0.284	0.294	0.769
Valency: Good × Gender match: Mismatch	0.056	0.154	− 0.246, 0.358	0.362	0.718
Valency: Bad × Gender match: Mismatch	− 0.045	0.145	− 0.33, 0.24	− 0.312	0.755

See analysis section for the model specification

**﻿Table 2:** Descriptives corresponding to significant main effects for first-party acceptability, third-party acceptability and prevalence. The *p* value was calculated from a one-sample Wilcoxon test with a chance level of 0.5

	*M*	*SE*	*p*
First-Party Acceptability			
Type Frequency			
high	0.642	0.019	< .001
low	0.612	0.019	< .001
Valency			
good	0.960	0.008	< .001
neutral	0.788	0.011	< .001
bad	0.132	0.011	< .001
Third-Party Acceptability			
Type Frequency			
high	0.722	0.012	< .001
low	0.621	0.013	< .001
Valency			
good	0.937	0.008	< .001
neutral	0.799	0.009	< .001
bad	0.280	0.013	< .001
Behavior prevalence			
Type Frequency			
high	0.709	0.011	< .001
low	0.445	0.012	< .001
Valency			
good	0.753	0.012	< .001
neutral	0.583	0.013	< .001
bad	0.396	0.015	< .001
Question Generality			
gendered	0.609	0.012	< .001
general	0.546	0.012	< .001

## Discussion

We asked whether learning about social groups relies on similar mechanisms as learning in other domains, focusing on the role of type frequency, that is, the number of distinct exemplars of a property. Participants read stories about behaviors in imaginary cities that were either frequently performed by a single actor (low type frequency), or rarely by multiple actors (high type frequency). High type frequency behaviors were deemed more morally acceptable, both in general and for the habitants of the imaginary city. However, in the case of first-party acceptability, the type frequency predominantly affected evaluations of intrinsically neutral (rather than good or bad) behaviors. This might be because participants presumably hold stronger beliefs about their own moral evaluations than about the evaluations by third parties; as a result, they might be more willing to update beliefs about third-party evaluations based on external information, and update beliefs about their own evaluations primarily when those beliefs are relatively weak to begin with (i.e., for neutral behaviors). Be that as it may, neutral behaviors can thus acquire moral valency simply by virtue of being performed by multiple actors, while moral evaluations of behaviors with an intrinsic valency seem less affected by the type frequency of the behaviors. In contrast, third-party acceptability judgements tracked the behaviors’ type frequency irrespective of the behaviors’ intrinsic valency.

In line with the predictions of an ideal observer model, high type frequency behaviors were also deemed to be more prevalent in the cities than behaviors that had been observed equally often. Observers thus use type frequency as a cue to the moral acceptability of a behavior and to its typicality in a group.

Participants also rated morally good behaviors as more prevalent than neutral behaviors and bad behaviors. This optimism about human nature might reflect a general tendency to treat positive information as more prevalent (Fiske, 1980; Unkelbach, Fiedler, Bayer, Stegmüller, & Danner, 2008); it might also reflect the greater tendency to attribute goodness (rather than badness) to an internal, unchanging “essence” of an actor (Heiphetz, 2019).^4^

Participants also thought that actors sharing a gender were more likely to engage in the same kinds of behaviors, though the match between the participants’ gender and those of the actors did not seem to affect the results.

Taken together, these results suggest that, just as in the case of language acquisition, type frequency helps observers learn moral conventions as well as typical behavior in other groups. Social factors (e.g., conformity) are unlikely to account for these results, simply because participants did not interact with the groups in the scenarios, and had no prior associations about these groups. In contrast, these results are compatible with the view that learners have epistemic biases when making inferences about other groups (Kim & Spelke, 2020); if so, they might use type frequency as a cue for selecting reliable or diagnostic information.^5^

The results have implications for the formation and reduction of stereotypes. For example, views about groups are more likely to be affected by exposure to numerous representatives of a group than by few but highly prominent representatives (e.g., media personalities); the latter represents a low type frequency/high token frequency situation, while the former represents a high type frequency/low token frequency situation. That being said, a high token frequency might still be important for memory retrieval; for example, and as mentioned above, a sufficient token frequency might be important for remembering specific dispositions (Endress & Hauser, 2011) or due to the availability heuristic (Tversky & Kahneman, 1973).

Conversely, a greater number of less prominent counter-stereotypic individuals might be more effective at combating stereotypes than a small number of highly prominent counter-stereotypic individuals. For example, a greater number of less prominent female scientist might lead to a greater reduction in gender-science stereotypes than fewer highly visible star scientists (e.g., Nobel laureates), though many other factors likely contribute to the success of role-model as well (Olsson & Martiny, 2018). The current results suggest that, to change group representations, role models need to occur with sufficient type frequency.

Taken together, these results suggest that basic learning principles from other domains might inform the formation of social representations, calling for a greater integration of language acquisition research with social learning.

## Supplementary Information

ESM 1 (PDF 394 KB)

## Data Availability

The code and data is available at 10.25383/city.14892078.

## Footnotes

1 Other authors proposed that observers might learn moral rules by inferring generative causal models of social interactions using “domain-general” mechanisms (Railton, 2017). Such models “imagine” a rich social situation to make predictions about different outcomes, using mechanisms shared across different domains rather than specifically social mechanisms. While we agree that the computational principles investigated here are available in several domains, such cross-domain availability does not imply the domain-generality of the underlying computations (Endress, 2019) Further, the current data do not speak to the richness of the internal models learners entertain; we only attempt to elucidate some of the computational principles used for the learning of socially relevant information.

2 McHugh et al., (2021) use the terms *type* and *token* differently from how they are used here. In their terminology, types are categories (e.g., good vs. bad) and tokens are members of these categories (e.g., helping a stranger vs. hurting a stranger). In our usage, types are the distinct individuals about whom predicates are true (e.g., the individuals who help a stranger), and tokens are the distinct observations of the predicate being true for some individual.

3 In the current experiments, we focus on the role of type frequency, both because this form of frequency drives generalizations in language acquisition, and because token frequency would be more difficult to study in the current design. In fact, the token frequency of an item determines how well it is remembered (eg., Bybee, 1995; Dabrowska & Szczerbiński, 2006; Endress & Hauser^2011^). Just informing participants about how often an event occurred thus does not have the same memory implications as actually varying the frequency of *experiencing* an event. After all, unlike with sequentially encountered events, the memory strength of an event presumably does not depend on its token frequency if all occurrences happen at once while participants are reading the scenario, and if the token frequency is just a number participants read together with the scenario.

4 While people often hold negative views about out-groups, this negativity bias might reflect the relative diagnosticity of negative information due to its rarity (Alves, Koch, & Unkelbach, 2018). Since the actions in the scenarios were chosen to be unusual, they are likely salient in themselves, and would not lead to a negativity bias.

5 While the computations seem to be similar across language acquisition and social learning, this does not imply that they rely on the same, domain-general cognitive mechanisms; rather, they might reflect similar computational principles that exist independently for linguistic, social and other stimuli (Endress, 2019).
