# Supplementary material for: Generic learning mechanisms can drive social inferences: The role of type frequency
Source: Mem Cognit. 2022 Apr 14;50(8):1694–705. doi: 10.3758/s13421-022-01286-2 (PMC9768010; doi:10.3758/s13421-022-01286-2)
Supplement: Supplementary file 1 — (PDF 394 KB) [file 13421_2022_1286_MOESM1_ESM.pdf]

Supplementary Online Materials

SOM1 Posterior probability of the actor being typical of a population

We use Bayes' rule to calculate the posterior probability the hypotheses H_1 that the actor is typical of a population

$$P(H_1|B) = \frac{P(B|H_1)P(H_1)}{\sum_{H'} P(B|H')p(H')}$$

where the sum runs over all hypotheses (i.e., that the actor is typical of the population and that they are exceptions). With the simplifying assumptions that the prior probability of the hypotheses reflects the expectations about the frequency of exceptions and that behaviors are observed independently from one another, this becomes

$$
\begin{aligned}
P(H_1|B) &= \frac{P(H_1)}{\sum_{H'} P(B|H')p(H')} \times \\
&\quad (P(B|\text{Actor typical}, H_1)P(\text{Actor typical}|H_1)+ \\
&\quad P(B|\text{Actor exception}, H_1)P(\text{Actor exception}|H_1)) \\
&= \frac{1-\epsilon}{\sum_{H'} P(B|H')p(H')} \times (\alpha(1-\epsilon)+\alpha\epsilon)^T \\
&= \frac{(1-\epsilon)\alpha^T}{\sum_{H'} P(B|H')p(H')} \\
&= \frac{(1-\epsilon)\alpha^T}{(1-\epsilon)\alpha^T + \epsilon\left(\beta(1-\epsilon)+\alpha\epsilon\right)^T} \\
&= \frac{1}{1+\frac{\epsilon}{1-\epsilon}\left(\frac{\beta}{\alpha}(1-\epsilon)+\epsilon\right)^T} \\
&\approx 1-\epsilon\left(\frac{\beta}{\alpha}\right)^T
\end{aligned}
$$

where the last step is the Taylor expansion for small values of ϵ. The probability of the behavior being typical of the population exponentially converges to 1 as the type frequency increases.

SOM2 Detection of violations of normality

As shown in Table S1, a Shapiro-Wilk test detected several violations of normality when considering raw ratings. For first-person acceptability, Tukey's ladder suggests a λ of 1.525 and the Box-Cox transform one of 1.3. For third-person acceptability, Tukey's ladder suggests a λ of 1.65 and the Box-Cox transform one of 1.6. For behavior prevalence, ukey's ladder suggests a λ of 0.925 and the Box-Cox transform one of 1.

Table S1

Cells for which a Shapiro-Wilk was significant.

	Valency	Type Frequency	Question Generality	W	p.value	p<=.05
**First-person acceptability**						
pilot experiment	good	high		0.632	0.000	***
pilot experiment	neutral	high		0.952	0.002	**
pilot experiment	bad	high		0.863	0.000	***
pilot experiment	good	low		0.725	0.000	***
pilot experiment	bad	low		0.901	0.000	***
main experiment	good	high		0.462	0.000	***
main experiment	neutral	high		0.927	0.000	***
main experiment	bad	high		0.857	0.000	***
main experiment	good	low		0.524	0.000	***
main experiment	neutral	low		0.948	0.000	***
main experiment	bad	low		0.889	0.000	***
**Third-person acceptability**						
pilot experiment	good	high	gendered	0.813	0.000	***
pilot experiment	neutral	high	gendered	0.945	0.001	***
pilot experiment	bad	high	gendered	0.946	0.001	**
pilot experiment	good	low	gendered	0.900	0.000	***
pilot experiment	bad	low	gendered	0.967	0.022	*
pilot experiment	good	high	general	0.782	0.000	***
pilot experiment	neutral	high	general	0.953	0.002	**
pilot experiment	bad	high	general	0.961	0.009	**
pilot experiment	good	low	general	0.894	0.000	***
pilot experiment	neutral	low	general	0.961	0.008	**
pilot experiment	bad	low	general	0.953	0.003	**
main experiment	good	high	gendered	0.566	0.000	***
main experiment	neutral	high	gendered	0.873	0.000	***
main experiment	bad	high	gendered	0.959	0.000	***
main experiment	good	low	gendered	0.732	0.000	***
main experiment	neutral	low	gendered	0.919	0.000	***
main experiment	bad	low	gendered	0.942	0.000	***
main experiment	good	high	general	0.606	0.000	***
main experiment	neutral	high	general	0.928	0.000	***
main experiment	bad	high	general	0.956	0.000	***
main experiment	good	low	general	0.735	0.000	***
main experiment	neutral	low	general	0.935	0.000	***
main experiment	bad	low	general	0.934	0.000	***
**Behavior prevalence**						
pilot experiment	good	high	gendered	0.909	0.000	***
pilot experiment	neutral	high	gendered	0.954	0.003	**
pilot experiment	bad	high	gendered	0.959	0.007	**
pilot experiment	bad	low	gendered	0.956	0.004	**
pilot experiment	good	high	general	0.935	0.000	***
pilot experiment	neutral	high	general	0.971	0.042	*
pilot experiment	bad	high	general	0.968	0.026	*
pilot experiment	good	low	general	0.962	0.010	**
pilot experiment	bad	low	general	0.953	0.003	**
main experiment	good	high	gendered	0.868	0.000	***
main experiment	neutral	high	gendered	0.956	0.000	***
main experiment	bad	high	gendered	0.966	0.001	**
main experiment	good	low	gendered	0.970	0.003	**
main experiment	neutral	low	gendered	0.969	0.002	**
main experiment	bad	low	gendered	0.963	0.001	***
main experiment	good	high	general	0.927	0.000	***
main experiment	neutral	high	general	0.971	0.004	**
main experiment	bad	high	general	0.980	0.036	*
main experiment	good	low	general	0.972	0.005	**
main experiment	neutral	low	general	0.977	0.016	*
main experiment	bad	low	general	0.956	0.000	***

SOM3 Pilot experiment

The pilot experiment was identical to the main experiment, except that participants were recruited from the Psychology Study Pool at City, University of London. Given that the sample was almost exclusively female, we removed the few male participants for consistency.

SOM3.1 First-party acceptability

The first analysis focused on first-party acceptability, that is, the degree to which the participants *themselves* considered a behavior morally acceptable. The results are shown in Tables S2 and S3 as well as Figure S1.

Good behaviors were rated as much more acceptable than neutral behaviors, which were rated much more acceptable than bad behaviors. In other words, the participants' first-party acceptability judgements validated our valency manipulation.

Critically, ratings were higher in the high type frequency condition than in the low type frequency condition, suggesting that the number of *different* individuals performing a behavior (rather than the raw frequency of a behavior) affects its moral acceptability even when observers just read about hypothetical scenarios. There was an interaction between *Type Frequency* and *Valency*. Follow-up GLMMs revealed that the effect of *Type Frequency* was more pronounced for neutral scenarios than for other scenarios.

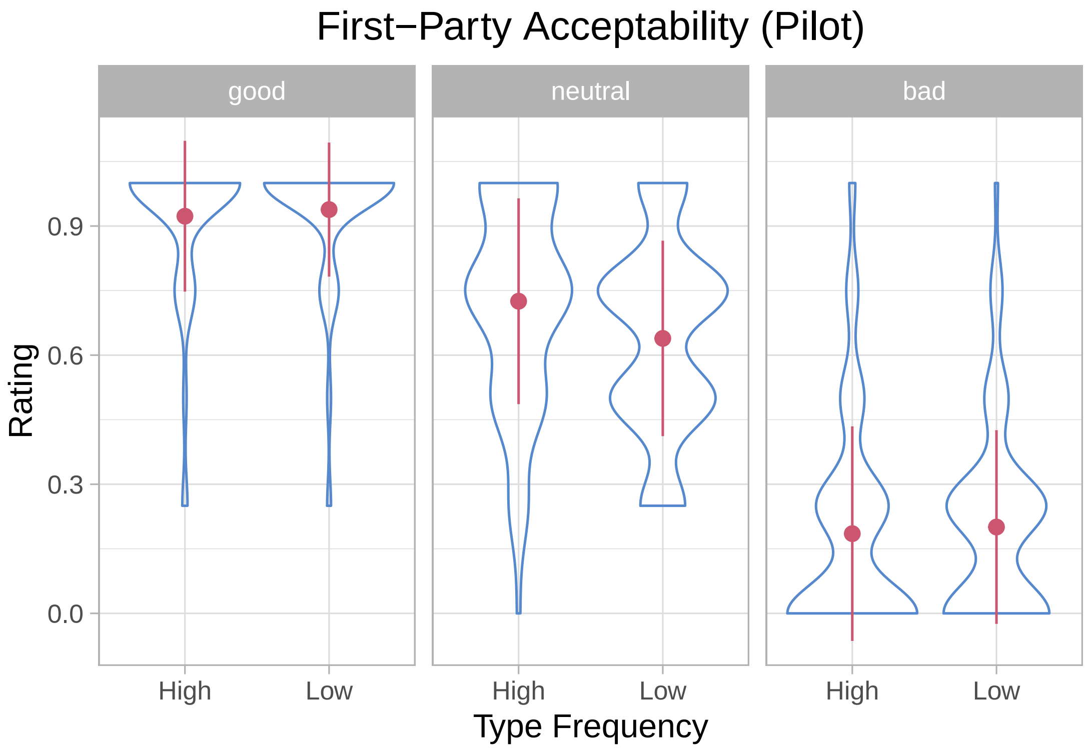

Figure S1. First-party acceptability as a function of Valency and Type Frequency in the pilot experiment. The contours represent the distribution of responses, the dots the sample averages and the errors bars the standard deviations.

SOM3.2 Third-party acceptability

We next explored the determinants of third-party acceptability, that is, the perceived moral acceptability of behaviors for other habitants of the imaginary city in a scenario. As mentioned above, we asked this question both in a gendered and a non-gendered way. That is, participants were asked (1) how acceptable a behavior would be *for other (wo)men* in the imaginary city when the behavior was performed by a (wo)man, and (2) how acceptable the behavior would be for habitants of the imaginary city *in general.*

The results are shown in Tables S2 and S3 as well as in Figure S2.

As for first-party acceptability, third-party acceptability ratings were higher for good behaviors than for neutral behaviors, and higher for neutral behaviors than for bad behaviors. Critically, third-party acceptability ratings were higher in the high type frequency condition than in the low type frequency condition, suggesting that participants believe that the number of *different* individuals performing a behavior (rather than the raw frequency of a behavior) reflects its moral acceptability in a community.

There was a marginal effect of *Question Generality*, suggesting that the ratings were marginally higher for the gendered questions rather than the general one. While the estimate had the same sign in the main experiment, it was not statistically reliable.

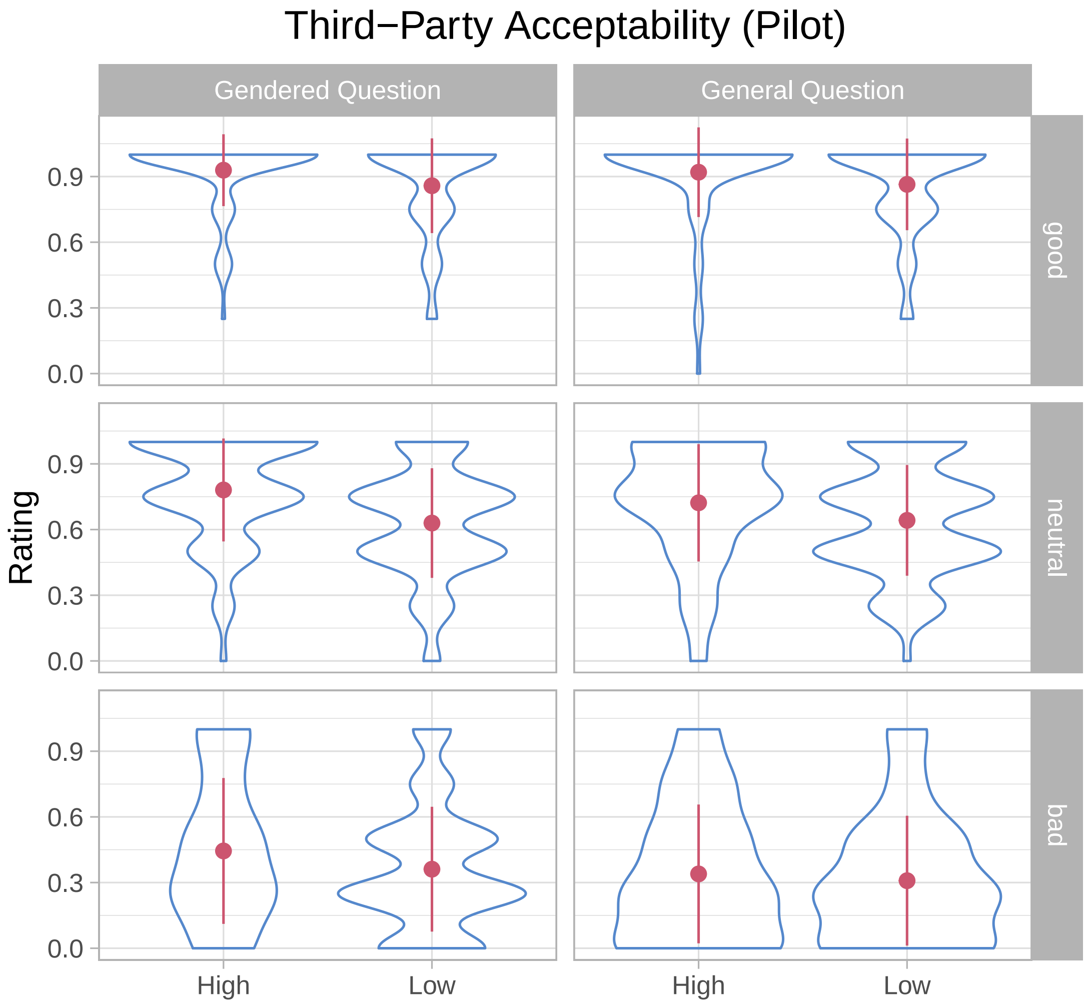

Figure S2. Third-party acceptability as a function of Valency and Type Frequency for the pilot experiment. The contours represent the distribution of responses, the dots the sample averages and the errors bars the standard deviations.

SOM3.3 Behavior prevalence

We finally explored the determinants of behavior prevalence, that is, the belief that other members of a group (i.e., other habitants of the city in the scenario) would engage in a behavior. As mentioned above, we asked this question both in a gendered and a non-gendered way. That is, participants were asked (1) how likely *other (wo)men* in the imaginary city would be to perform a behavior when it was performed by a (wo)man, and (2) how likely people in the imaginary city *in general* would be to perform the behavior.

The results are shown in Tables S2 and S3 as well as in Figure S3.

The prevalence of good behaviors was rated higher than that of neutral behaviors, which was rated higher than that of bad behaviors. Critically, the prevalence was rated higher for high type frequency behaviors than for low type frequency behaviors, suggesting that participants use the number of *different* individuals performing a behavior (rather than its raw frequency) as an indicator of behavior prevalence.

Finally, the prevalence ratings were somewhat higher for the gendered question than for the general question, suggesting that participants were more likely to generalize a behavior within genders than across genders. This effect was marginally less pronounced in the low type frequency condition, presumably because prevalence ratings are lower in the low type frequency condition to begin with.

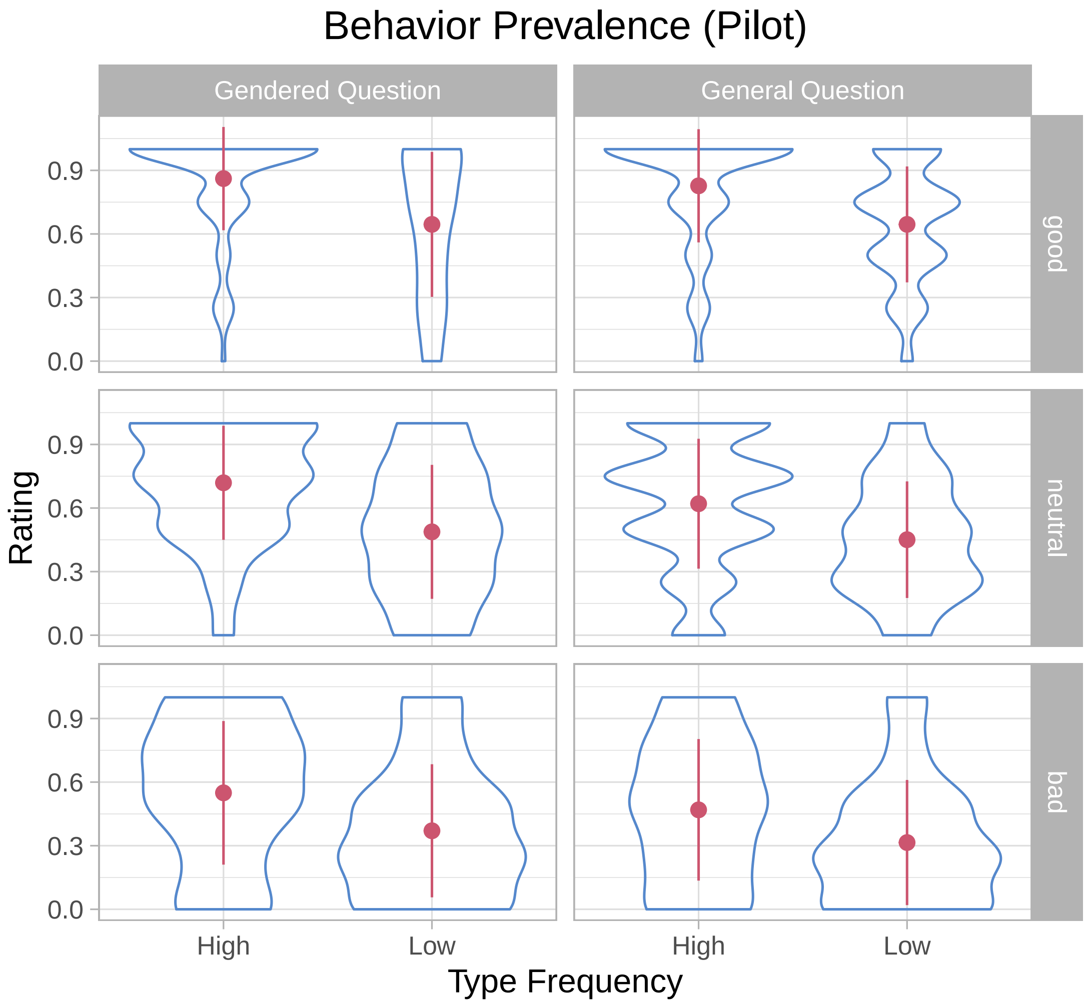

Figure S3. Behavior prevalence as a function of Valency, Type Frequency and Question Generality (gendered vs. general question) for the pilot experiment. The contours represent the distribution of responses, the dots the sample averages and the errors bars the standard deviations.

Table S2

Results of generalized linear mixed models of the trial-by-trial data for first-party acceptability, third-party acceptability and behavior prevalence for the pilot experiment. See analysis section for the model specification.

Effect	Estimate	*SE*	*CI*	*t*	*p*
**First-party acceptability - Overall GLMM**					
Type Frequency: Low	-0.575	0.205	-0.977, -0.174	-2.810	0.005
Valency: Good	1.503	0.604	0.319, 2.69	2.489	0.013
Valency: Bad	-3.316	0.589	-4.47, -2.16	-5.634	0.000
Type Frequency: Low × Valency: Good	0.845	0.372	0.115, 1.58	2.269	0.023
Type Frequency: Low × Valency: Bad	0.671	0.300	0.0825, 1.26	2.235	0.025
**First-party acceptability - Neutral valency only**					
Type Frequency: Low	-0.612	0.210	-1.02, -0.2	-2.908	0.004
**First-party acceptability - Neutral valency exlcuded**					
Type Frequency: Low	0.276	0.308	-0.328, 0.88	0.895	0.371
Valency: Bad	-4.715	0.475	-5.65, -3.78	-9.919	0.000
Type Frequency: Low × Valency: Bad	-0.173	0.376	-0.91, 0.564	-0.460	0.646
**Third-party acceptability**					
Question Generality: General	-0.321	0.166	-0.647, 0.0052	-1.929	0.054
Type Frequency: Low	-0.878	0.165	-1.2, -0.554	-5.316	0.000
Valency: Good	1.356	0.420	0.533, 2.18	3.228	0.001
Valency: Bad	-1.871	0.393	-2.64, -1.1	-4.761	0.000
Question Generality: General × Type Frequency: Low	0.322	0.169	-0.00905, 0.654	1.906	0.057
Question Generality: General × Valency: Good	0.101	0.233	-0.355, 0.557	0.434	0.664
Question Generality: General × Valency: Bad	-0.251	0.189	-0.621, 0.119	-1.328	0.184
Type Frequency: Low × Valency: Good	-0.041	0.238	-0.509, 0.426	-0.174	0.862
Type Frequency: Low × Valency: Bad	0.395	0.190	0.0232, 0.767	2.082	0.037
**Behavior prevalence**					
Question Generality: General	-0.419	0.200	-0.811, -0.0275	-2.098	0.036
Type Frequency: Low	-1.335	0.173	-1.67, -0.997	-7.730	0.000
Valency: Good	1.158	0.375	0.423, 1.89	3.088	0.002
Valency: Bad	-1.039	0.359	-1.74, -0.335	-2.893	0.004
Gender Match: Mismatch	0.026	0.305	-0.571, 0.623	0.086	0.932
Question Generality: General × Type Frequency: Low	0.257	0.154	-0.0448, 0.559	1.669	0.095
Question Generality: General × Valency: Good	0.013	0.272	-0.519, 0.546	0.049	0.961
Question Generality: General × Valency: Bad	0.301	0.255	-0.198, 0.801	1.182	0.237
Type Frequency: Low × Valency: Good	-0.239	0.198	-0.626, 0.148	-1.208	0.227
Type Frequency: Low × Valency: Bad	0.208	0.182	-0.149, 0.565	1.141	0.254
Question Generality: General × Gender Match: Mismatch	-0.174	0.259	-0.682, 0.334	-0.671	0.502
Type Frequency: Low × Gender Match: Mismatch	0.273	0.155	-0.0317, 0.577	1.756	0.079
Valency: Good × Gender Match: Mismatch	-0.307	0.277	-0.85, 0.236	-1.108	0.268
Valency: Bad × Gender Match: Mismatch	0.320	0.257	-0.184, 0.824	1.243	0.214
Question Generality: General × Valency: Good × Gender Match: Mismatch	0.441	0.385	-0.314, 1.2	1.145	0.252
Question Generality: General × Valency: Bad × Gender Match: Mismatch	-0.524	0.361	-1.23, 0.184	-1.452	0.147

Table S3

Descriptives corresponding to significant main effects for the pilot experiment for first-party acceptability, third-party acceptability and behavior prevalence. The p value was calculated from a one-sample Wilcoxon test with a chance level of 0.5.

Condition	*M*	*SE*	*p*
**First-Party Acceptability - Type Frequency**			
high	0.611	0.025	< .001
low	0.593	0.024	< .001
**First-Party Acceptability - Valency**			
good	0.931	0.013	< .001
neutral	0.682	0.019	< .001
bad	0.193	0.019	< .001
**Third-Party Acceptability - Type Frequency**			
high	0.689	0.016	< .001
low	0.611	0.015	< .001
**Third-Party Acceptability - Valency**			
good	0.893	0.011	< .001
neutral	0.694	0.014	< .001
bad	0.363	0.017	< .001
**Behavior prevalence - Type Frequency**			
high	0.674	0.015	< .001
low	0.486	0.015	< .465
**Behavior prevalence - Valency**			
good	0.745	0.017	< .001
neutral	0.569	0.017	< .001
bad	0.426	0.018	< .001
**Behavior prevalence - Question Generality**			
gendered	0.605	0.016	< .001
general	0.555	0.015	< .001

SOM4 Descriptives for individual cells

Table S4

Descriptives for first-party acceptability in different cells for the main experiment (top) and the pilot experiment (bottom). The pilot experiment includes only female participants.

Valency	Type Frequency	*M*	*SE*
**Main experiment**			
good	high	3.81	0.039
good	low	3.78	0.038
neutral	high	3.31	0.044
neutral	low	3.19	0.040
bad	high	1.53	0.046
bad	low	1.49	0.038
**Pilot experiment**			
good	high	3.73	0.053
good	low	3.72	0.045
neutral	high	3.09	0.069
neutral	low	2.88	0.063
bad	high	1.68	0.079
bad	low	1.68	0.065

Table S5

Descriptives for third-party acceptability in different cells for the main experiment (top) and the pilot experiment (bottom). The pilot experiment includes only female participants.

Generality	Valency	Type Frequency	*M*	*SE*
**Main experiment**				
gendered	good	high	3.76	0.040
gendered	good	low	3.57	0.050
gendered	neutral	high	3.38	0.045
gendered	neutral	low	3.09	0.048
gendered	bad	high	2.23	0.066
gendered	bad	low	1.85	0.050
general	good	high	3.72	0.042
general	good	low	3.57	0.050
general	neutral	high	3.33	0.038
general	neutral	low	3.07	0.046
general	bad	high	2.06	0.060
general	bad	low	1.77	0.048
**Pilot experiment**				
gendered	good	high	3.57	0.056
gendered	good	low	3.37	0.061
gendered	neutral	high	3.16	0.063
gendered	neutral	low	2.83	0.061
gendered	bad	high	2.38	0.083
gendered	bad	low	2.16	0.070
general	good	high	3.57	0.059
general	good	low	3.42	0.059
general	neutral	high	3.10	0.063
general	neutral	low	2.85	0.056
general	bad	high	2.15	0.077
general	bad	low	2.04	0.072

Table S6

Descriptives for behavior prevalence in different cells for the main experiment (top) and the pilot experiment (bottom). The pilot experiment includes only female participants.

Generality	Valency	Type Frequency	*M*	*SE*
**Main experiment**				
gendered	good	high	3.43	0.048
gendered	good	low	2.89	0.053
gendered	neutral	high	3.11	0.050
gendered	neutral	low	2.47	0.050
gendered	bad	high	2.74	0.065
gendered	bad	low	2.14	0.051
general	good	high	3.22	0.050
general	good	low	2.78	0.050
general	neutral	high	2.83	0.041
general	neutral	low	2.39	0.048
general	bad	high	2.48	0.055
general	bad	low	1.98	0.045
**Pilot experiment**				
gendered	good	high	3.32	0.065
gendered	good	low	2.83	0.074
gendered	neutral	high	3.03	0.072
gendered	neutral	low	2.52	0.066
gendered	bad	high	2.72	0.076
gendered	bad	low	2.31	0.067
general	good	high	3.19	0.063
general	good	low	2.78	0.052
general	neutral	high	2.81	0.068
general	neutral	low	2.46	0.055
general	bad	high	2.45	0.066
general	bad	low	2.18	0.059

SOM5 Scenarios

The following list provides the scenarios used in the experiments. The difference between the counterbalancing conditions is which scenario occurred in the 1 person/10 repetitions (low type frequency) condition, and which scenario occurred in the 10 persons/1 repetition (high type frequency condition). Another two lists were prepared with male actors.

SOM5.1 Cover story

The following cover story preceded the presentation of the scenarios:

Noah's travel journal

Noah is studying journalism at university. An opportunity comes up where he decides to take a gap year to explore some countries and cities that have always interested him. He usually stays 10 days in each place. In each place, Noah makes some interesting observations.

SOM5.2 Counterbalancing Condition 1

SOM5.2.1 Bad behaviors.

- In Marjy, Noah saw the same woman park in a priority disabled parking space on each of the 10 days of his stay.
  - How likely are *women* in Marjy to park in priority disabled parking space?
  - How likely are people in Marjy *in general* to park in priority disabled parking space?
  - Would *women* in Marjy consider it morally acceptable to park priority disabled parking space?
  - Would people in Marjy *in general* consider it morally acceptable to park in disabled parking space?
  - Would *you* consider it morally acceptable to park in priority disabled parking space?
- In Bruff, Noah walked along the high street on each day of this stay. On each of the 10 occasions, he saw the same woman punch a police officer.

- How likely are *women* in Bruff to punch police officers?
- How likely are people in Bruff *in general* to punch police officers?
- Would *women* in Bruff consider it morally acceptable to punch police officers?
- Would people in Bruff *in general* consider it morally acceptable to punch police officers?
- Would *you* consider it morally acceptable to punch police officers?

- In Quai, Noah went to the cinema every evening; he saw the same woman skip the queue on each of his 10 visits.
  - How likely are *women* in Quai to skip a queue?
  - How likely are people in Quai *in general* to skip a queue?
  - Would *women* in Quai consider it morally acceptable to skip a queue?
  - Would people in Quai *in general* consider it morally acceptable to skip a queue?
  - Would *you* consider it morally acceptable to skip a queue?
- In Sephy, Noah walked to the train station every morning. On all ten occasions, he saw the same woman cycle past a red traffic light on her bike.
  - How likely are *women* in Sephy to cycle past a red light?
  - How likely are people in Sephy *in general* to cycle past a red light?
  - Would *women* in Sephy consider it morally acceptable to cycle past a red light?
  - Would people in Sephy *in general* consider it morally acceptable to cycle past a red light?
  - Would *you* consider it morally acceptable to cycle past a red light?
- During Noah's stay in a village called Freezington, Noah passed by a farm every day. On each of the ten days, he saw a different woman pillaging corn from a field.
  - How likely are *women* in Freezington to pillage corn from a field?
  - How likely are people in Freezington *in general* to pillage corn from a field?
  - Would *women* in Frezzington consider it morally acceptable to pillage corn from a field?
  - Would people in Freezington *in general* consider it morally acceptable to pillage corn from a field?
  - Would *you* consider it morally acceptable to pillage corn from a field?
- During Noah's stay in Glistinir, he took the bus every day. On all 10 days while waiting at the bus stop, Noah saw a different woman lowering her car's window to shout at the driver next to her.
  - How likely are *women* in Glistinir to shout at other drivers?

  - How likely are people in Glistinir *in general* to shout at other drivers?
  - Would *women* in Glistinir consider it morally acceptable to shout at other drivers?
  - Would people in Glistinir *in general* consider it morally acceptable to shout at other drivers?
  - Would *you* consider it morally acceptable to shout at other drivers?
- In Zlefwood, Noah walked along the canal every day and saw a different woman littering in the canals on each of his ten walks.

  - How likely are *women* in Zlefwood to litter in canals?
  - How likely are people in Zlefwood *in general* to litter in canals?
  - Would *women* in Zlefwood consider it morally acceptable to litter in canals?
  - Would people in Zlefwood *in general* consider it morally acceptable to litter in canals?
  - Would *you* consider it morally acceptable to litter in canals?
- In Cluakpus, Noah went to the bakery every morning and saw a different woman smoking marijuana in front of the bakery on each of his 10 visits.

  - How likely are *women* in Cluakpus to smoke marijuana?
  - How likely are people in Cluakpus *in general* to smoke marijuana?
  - Would *women* in Cluakpus consider it morally acceptable to smoke marijuana?
  - Would people in Cluakpus *in general* consider it morally acceptable to smoke marijuana?
  - Would *you* consider it morally acceptable to smoke marijuana?

SOM5.2.2 Good behaviors.

- In Therntuthern, Noah purposely dropped his wallet on the street every day, and, on all 10 occasions the same woman would return the wallet by calling him and handing it over.

  - How likely are *women* in Therntuthern to return wallets?
  - How likely are people in Therntuthern *in general* to return wallets?
  - Would *women* in Therntuthern consider it morally acceptable to return a dropped wallet?
  - Would people in Therntuthern *in general* consider it morally acceptable to return a dropped wallet?
  - Would *you* consider it morally acceptable to return a dropped wallet?
- In Wrury, Noah got lost every day. He happened to ask the same woman for directions on the 10 occasions, and he always received a helpful answer.

- How likely are *women* in Wrury to give directions?
- How likely are people in Wrury *in general* to give directions?
- Would *women* in Wrury consider it morally acceptable to give directions?
- Would people in Wrury *in general* consider it morally acceptable to give directions?
- Would *you* consider it morally acceptable to give directions?

- In Gralens, Noah passed in front of the retirement home every day. There, he saw the same woman help an elderly person across the road on all 10 instances.

  - How likely are *women* in Gralens to help the elderly cross a road?
  - How likely are people in Gralens *in general* to help the elderly cross a road?
  - Would *women* in Gralens consider it morally acceptable to help the elderly cross a road?
  - Would people in Gralens *in general* consider it morally acceptable to help the elderly cross a road?
  - Would *you* consider it morally acceptable to help the elderly cross a road?
- Noah visited Vali during fledgling season. He passed through the main square every day, and on each of the 10 occasions, Noah saw the same woman run to save a baby bird from being run over by a car.

  - How likely are *women* in Vali to save baby birds?
  - How likely are people in Vali *in general* to save baby birds?
  - Would *women* in Vali consider it morally acceptable to save baby birds?
  - Would people in Vali *in general* consider it morally acceptable to save baby birds?
  - Would *you* consider it morally acceptable to save baby birds?
- In Styl, Noah used the bus every day. On each of ten occasions, he saw a different woman offer her seat to a pregnant lady.

  - How likely are *women* in Styl to offer their seat to pregnant women?
  - How likely are people in Styl *in general* to offer their seat to pregnant women?
  - Would *women* in Styl consider it morally acceptable to offer their seat to pregnant women?
  - Would people in Styl *in general* consider it morally acceptable to offer their seat to pregnant women?
  - Would *you* consider it morally acceptable to offer their seat to pregnant women?
- In Pastel, Noah visited the local café every morning. On each of his 10 visits,

he saw a different woman buying coffee for her colleagues at her workplace.

  - How likely are *women* in Pastel to buy coffee for their colleagues?
  - How likely are people in Pastel *in general* to buy coffee for their colleagues?
  - Would *women* in Pastel consider it morally acceptable to buy coffee for their colleagues?
  - Would people in Pastel *in general* consider it morally acceptable to buy coffee for their colleagues?
  - Would *you* consider it morally acceptable to buy coffee for their colleagues?

- In Tiare, Noah went for a jog in a park on each day of his visit. On all ten occasions, he saw a different woman on the playground giving her own and other children fruit when they got hungry.

  - How likely are *women* in Tiare to give fruit to other hungry children?
  - How likely are people in Tiare *in general* to give fruit to other hungry children?
  - Would *women* in Tiare consider it morally acceptable to give fruit to other hungry children?
  - Would people in Tiare *in general* consider it morally acceptable to give fruit to other hungry children?
  - Would *you* consider it morally acceptable to give fruit to other hungry children?

- During Noah's stay in Windon, Noah took went to the local train station every day, and saw a different woman putting money in a bucket for a children's charity on all 10 days.

  - How likely are *women* in Windon to donate to a children's charity?
  - How likely are people in Windon *in general* to donate to a children's charity?
  - Would *women* in Windon consider it morally acceptable to donate to a children's charity?
  - Would people in Windon *in general* consider it morally acceptable to donate to a children's charity?
  - Would *you* consider it morally acceptable to donate to a a children's charity?

SOM5.2.3 Neutral behaviors.

- In Qrita, Noah saw the same woman at the bus stop every day of his 10 day stay and she would always wear sunglasses.

  - How likely are *women* in Qrita to wear sunglasses?
  - How likely are people in Qrita *in general* to wear sunglasses?
  - Would *women* in Qrita consider it morally acceptable to wear sunglasses?
  - Would people in Qrita *in general* consider it morally acceptable to wear sunglasses?
  - Would *you* consider it morally acceptable to wear sunglasses?
- In Krark, Noah went to the harbor every day and saw the same woman walking with her swimsuit on the street on all 10 days.
  - How likely are *women* in Krark to wear swimsuits on the street?
  - How likely are people in Krark *in general* to wear swimsuits on the street?
  - Would *women* in Krark consider it morally acceptable to wear swimsuits on the street?
  - Would people in Krark *in general* consider it morally acceptable to wear swimsuits on the street?
  - Would *you* consider it morally acceptable to wear swimsuits on the street?
- In Caco, Noah was passing through the park every day and saw the same woman doing acrobatics on all 10 times.
  - How likely are *women* in Caco to do acrobatics?
  - How likely are people in Caco *in general* to do acrobatics?
  - Would *women* in Caco consider it morally acceptable to do acrobatics?
  - Would people in Caco *in general* consider it morally acceptable to do acrobatics?
  - Would *you* consider it morally acceptable to do acrobatics?
- In Elder, Noah passed through the train station every day and saw the same woman pantomiming on each of his 10 visits.
  - How likely are *women* in Elder to pantomime?
  - How likely are people in Elder *in general* to pantomime?
  - Would *women* in Elder consider it morally acceptable to pantomime?
  - Would people in Elder *in general* consider it morally acceptable to pantomime?
  - Would *you* consider it morally acceptable to pantomime?
- In Yooley, Noah had breakfast in the cafeteria of the newspaper agency every day. On all 10 days, he saw a different woman arriving at work by bike.
  - How likely are *women* in Yooley to bike to work?
  - How likely are people in Yooley *in general* to bike to work?
  - Would *women* in Yooley consider it morally acceptable to bike to work?

  - Would people in Yooley *in general* consider it morally acceptable to bike to work?
  - Would *you* consider it morally acceptable to bike to work?
- In Kurk, Noah went to the local pub every evening and saw a different woman drinking a glass of wine on each of the 10 evenings.

  - How likely are *women* in Kurk to drink wine?
  - How likely are people in Kurk *in general* to drink wine?
  - Would *women* in Kurk consider it morally acceptable to drink wine?
  - Would people in Kurk *in general* consider it morally acceptable to drink wine?
  - Would *you* consider it morally acceptable to drink wine?
- Noah found himself in Aserad. On each of the 10 days during Noah's stay in Aserad, he took the bus to the city center and saw a different woman running alongside cars.

  - How likely are *women* in Aserad to run alongside cars?
  - How likely are people in Aserad *in general* to run alongside cars?
  - Would *women* in Aserad consider it morally acceptable to run alongside cars?
  - Would people in Aserad *in general* consider it morally acceptable to run alongside cars?
  - Would *you* consider it morally acceptable to run alongside cars?
- During Noah's stay in Ozmendir, Noah passed in front of the post office every day. On each of the 10 days, he saw a different woman eating raw broccoli for lunch.

  - How likely are *women* in Ozmendir to eat raw broccoli?
  - How likely are people in Ozmendir *in general* to eat raw broccoli?
  - Would *women* in Ozmendir consider it morally acceptable to eat raw broccoli?
  - Would people in Ozmendir *in general* consider it morally acceptable to eat raw broccoli?
  - Would *you* consider it morally acceptable to eat raw broccoli?

SOM5.3 Counterbalancing Condition 2

SOM5.3.1 Bad behaviors.

- In Marjy, Noah saw a different woman park in a priority disabled parking spot on each of the 10 days of his stay.

- How likely are *women* in Marjy to park in priority disabled parking space?
- How likely are people in Marjy *in general* to park in priority disabled parking space?
- Would *women* in Marjy consider it morally acceptable to park priority disabled parking space?
- Would people in Marjy *in general* consider it morally acceptable to park in disabled parking space?
- Would *you* consider it morally acceptable to park in priority disabled parking space?

• In Bruff, Noah walked along the high street on each day of this stay. On each of the 10 occasions, he saw a different woman punch a police officer.

- How likely are *women* in Bruff to punch police officers?
- How likely are people in Bruff *in general* to punch police officers?
- Would *women* in Bruff consider it morally acceptable to punch police officers?
- Would people in Bruff *in general* consider it morally acceptable to punch police officers?
- Would *you* consider it morally acceptable to punch police officers?

• In Quai, Noah went to the cinema every evening, and a different woman skipped the queue on each of his 10 visits.

- How likely are *women* in Quai to skip a queue?
- How likely are people in Quai *in general* to skip a queue?
- Would *women* in Quai consider it morally acceptable to skip a queue?
- Would people in Quai *in general* consider it morally acceptable to skip a queue?
- Would *you* consider it morally acceptable to skip a queue?

• In Sephy, Noah walked to the train station every morning. On all ten occasions, he saw the same woman cycle past a red traffic light on her bike.

- How likely are *women* in Sephy to cycle past a red light?
- How likely are people in Sephy *in general* to cycle past a red light?
- Would *women* in Sephy consider it morally acceptable to cycle past a red light?
- Would people in Sephy *in general* consider it morally acceptable to cycle past a red light?
- Would *you* consider it morally acceptable to cycle past a red light?

• During Noah's stay in a village called Freezington, Noah passed by a farm every day. On each of the ten days, he saw the same woman pillaging corn from a field.

  - How likely are *women* in Freezington to pillage corn from a field?
  - How likely are people in Freezington *in general* to pillage corn from a field?
  - Would *women* in Frezzington consider it morally acceptable to pillage corn from a field?
  - Would people in Freezington *in general* consider it morally acceptable to pillage corn from a field?
  - Would *you* consider it morally acceptable to pillage corn from a field?
- During Noah's stay in Glistinir, he took the bus every day. On all 10 days while waiting at the bus stop, Noah saw the same woman lowering her car's window to shout at the car next to her.

  - How likely are *women* in Glistinir to shout at other drivers?
  - How likely are people in Glistinir *in general* to shout at other drivers?
  - Would *women* in Glistinir consider it morally acceptable to shout at other drivers?
  - Would people in Glistinir *in general* consider it morally acceptable to shout at other drivers?
  - Would *you* consider it morally acceptable to shout at other drivers?
- In Zlefwood, Noah walked along the canal every day and saw the same woman littering in the canals on each of his ten walks.

  - How likely are *women* in Zlefwood to litter in canals?
  - How likely are people in Zlefwood *in general* to litter in canals?
  - Would *women* in Zlefwood consider it morally acceptable to litter in canals?
  - Would people in Zlefwood *in general* consider it morally acceptable litter in canals?
  - Would *you* consider it morally acceptable to litter in canals?
- In Cluakpus, Noah went to the bakery every morning and saw the same woman smoking marijuana in front of the bakery on each of his 10 visits.

  - How likely are *women* in Cluakpus to smoke marijuana
  - How likely are people in Cluakpus *in general* to smoke marijuana?
  - Would *women* in Cluakpus consider it morally acceptable to smoke marijuana?
  - Would people in Cluakpus *in general* consider it morally acceptable to smoke marijuana?
  - Would *you* consider it morally acceptable to smoke marijuana?

SOM5.3.2 Good behaviors.

- In Therntuthern, Noah purposely dropped his wallet on the street every day,

and, on all 10 occasions a different woman would return the wallet by calling him and handing it over.

  - How likely are *women* in Therntuthern to return wallets?
  - How likely are people in Therntuthern *in general* to return wallets?
  - Would *women* in Therntuthern consider it morally acceptable to return a dropped wallet?
  - Would people in Therntuthern *in general* consider it morally acceptable to return a dropped wallet?
  - Would *you* consider it morally acceptable to return a dropped wallet?
- In Wrury, Noah got lost every day. He happened to ask a different woman for directions on the 10 occasions, and he always received a helpful answer.
  - How likely are *women* in Wrury to give directions?
  - How likely are people in Wrury *in general* to give directions?
  - Would *women* in Wrury consider it morally acceptable to give directions?
  - Would people in Wrury *in general* consider it morally acceptable to give directions?
  - Would *you* consider it morally acceptable to give directions?
- In Gralens, Noah passed in front of the retirement home every day. There, he saw a different woman help an elderly person across the road on all 10 instances.
  - How likely are *women* in Gralens to help the elderly cross a road?
  - How likely are people in Gralens *in general* to help the elderly cross a road?
  - Would *women* in Gralens consider it morally acceptable to help the elderly cross a road?
  - Would people in Gralens *in general* consider it morally acceptable to help the elderly cross a road?
  - Would *you* consider it morally acceptable to help the elderly cross a road?
- Noah visited Vali during fledgling season. He passed through the main square every day, and on each of the 10 occasions, Noah saw a different woman run to save a baby bird from being run over by a car.
  - How likely are *women* in Vali to save baby birds?
  - How likely are people in Vali *in general* to save baby birds?
  - Would *women* in Vali consider it morally acceptable to save baby birds?
  - Would people in Vali *in general* consider it morally acceptable to save baby birds?
  - Would *you* consider it morally acceptable to save baby birds?

- In Styl, Noah used the bus every day. On each of ten occasions, he saw the same woman offer her seat to a pregnant lady.
  - How likely are *women* in Styl to offer their seat to pregnant women?
  - How likely are people in Styl *in general* to offer their seat to pregnant women?
  - Would *women* in Styl consider it morally acceptable to offer their seat to pregnant women?
  - Would people in Styl *in general* consider it morally acceptable to offer their seat to pregnant women?
  - Would *you* consider it morally acceptable to offer their seat to pregnant women?
- In Pastel, Noah visited the local café every morning. On each of his 10 visits, he saw the same woman buying coffee for her colleagues at her workplace.
  - How likely are *women* in Pastel to buy coffee for their colleagues?
  - How likely are people in Pastel *in general* to buy coffee for their colleagues?
  - Would *women* in Pastel consider it morally acceptable to buy coffee for their colleagues?
  - Would people in Pastel *in general* consider it morally acceptable to buy coffee for their colleagues?
  - Would *you* consider it morally acceptable to buy coffee for their colleagues?
- In Tiare, Noah went for a jog in a park on each day of his visit. On all ten occasions, he saw the same woman on the playground giving her own and other children fruit when they got hungry.
  - How likely are *women* in Tiare to give fruit to other hungry children?
  - How likely are people in Tiare *in general* to give fruit to other hungry children?
  - Would *women* in Tiare consider it morally acceptable to give fruit to other hungry children?
  - Would people in Tiare *in general* consider it morally acceptable to give fruit to other hungry children?
  - Would *you* consider it morally acceptable to give fruit to other hungry children?
- During Noah's stay in Windon, Noah took went to the local train station every day, and saw the same woman putting money in a bucket for a children's charity on all 10 days.
  - How likely are *women* in Windon to donate to a children's charity?
  - How likely are people in Windon *in general* to donate to a children's

charity?
  - Would *women* in Windon consider it morally acceptable to donate to a children's charity?
  - Would people in Windon *in general* consider it morally acceptable to donate to a children's charity?
  - Would *you* consider it morally acceptable to donate to a children's charity?

SOM5.3.3 Neutral behaviors.

- In Qrita, Noah saw a different woman at the bus stop every day of his 10 day stay and they would all wear sunglasses.
  - How likely are *women* in Qrita to wear sunglasses?
  - How likely are people in Qrita *in general* to wear sunglasses?
  - Would *women* in Qrita consider it morally acceptable to wear sunglasses?
  - Would people in Qrita *in general* consider it morally acceptable to wear sunglasses?
  - Would *you* consider it morally acceptable to wear sunglasses?
- In Krark, Noah went to the harbor every day and saw a different woman walking with her swimsuit on the street on all 10 days.
  - How likely are *women* in Krark to wear swimsuits on the street?
  - How likely are people in Krark *in general* to wear swimsuits on the street?
  - Would *women* in Krark consider it morally acceptable to wear swimsuits on the street?
  - Would people in Krark *in general* consider it morally acceptable to wear swimsuits on the street?
  - Would *you* consider it morally acceptable to wear swimsuits on the street?
- In Caco, Noah was passing through the park every day and saw a different woman doing acrobatics on all 10 times.
  - How likely are *women* in Caco to do acrobatics?
  - How likely are people in Caco *in general* to do acrobatics?
  - Would *women* in Caco consider it morally acceptable to do acrobatics?
  - Would people in Caco *in general* consider it morally acceptable to do acrobatics?
  - Would *you* consider it morally acceptable to do acrobatics?
- In Elder, Noah passed through the train station every day and saw a different woman pantomiming on each of his 10 visits.

  - How likely are *women* in Elder to pantomime?
  - How likely are people in Elder *in general* to pantomime?
  - Would *women* in Elder consider it morally acceptable to pantomime?
  - Would people in Elder *in general* consider it morally acceptable to pantomime?
  - Would *you* consider it morally acceptable to pantomime?
- In Yooley, Noah had breakfast in the cafeteria of the newspaper agency every day. On all 10 days, he saw the same woman arriving at work by bike.
  - How likely are *women* in Yooley to bike to work?
  - How likely are people in Yooley *in general* to bike to work?
  - Would *women* in Yooley consider it morally acceptable to bike to work?
  - Would people in Yooley *in general* consider it morally acceptable to bike to work?
  - Would *you* consider it morally acceptable to bike to work?
- In Kurk, Noah went to the local pub every evening and saw the same woman drinking a glass of wine on each of the 10 evenings.
  - How likely are *women* in Kurk to drink wine?
  - How likely are people in Kurk *in general* to drink wine?
  - Would *women* in Kurk consider it morally acceptable to drink wine?
  - Would people in Kurk *in general* consider it morally acceptable to drink wine?
  - Would *you* consider it morally acceptable to drink wine?
- Noah found himself in Aserad. On each of the 10 days during Noah's stay in Aserad, he took the bus to the city center and saw the same woman running alongside cars.
  - How likely are *women* in Aserad to run alongside cars?
  - How likely are people in Aserad *in general* to run alongside cars?
  - Would *women* in Aserad consider it morally acceptable to run alongside cars?
  - Would people in Aserad *in general* consider it morally acceptable to run alongside cars?
  - Would *you* consider it morally acceptable to run alongside cars?
- During Noah's stay in Ozmendir, Noah passed in front of the post office every day. On each of the 10 days, he saw the same woman eating raw broccoli for lunch.
  - How likely are *women* in Ozmendir to eat raw broccoli?
  - How likely are people in Ozmendir *in general* to eat raw broccoli?
  - Would *women* in Ozmendir consider it morally acceptable to eat raw broccoli?

- Would people in Ozmendir *in general* consider it morally acceptable to eat raw broccoli?
- Would *you* consider it morally acceptable to eat raw broccoli?
